# Co and Fe Codoped WO_2.72_ as Alkaline‐Solution‐Available Oxygen Evolution Reaction Catalyst to Construct Photovoltaic Water Splitting System with Solar‐To‐Hydrogen Efficiency of 16.9%

**DOI:** 10.1002/advs.201900465

**Published:** 2019-07-11

**Authors:** Huayu Chen, Lizhu Song, Shuxin Ouyang, Jianbo Wang, Jun Lv, Jinhua Ye

**Affiliations:** ^1^ TJU‐NIMS International Collaboration Laboratory School of Materials Science and Engineering Tianjin University Tianjin 300072 China; ^2^ College of Chemistry Central China Normal University Wuhan 430079 China; ^3^ School of Electronic Science and Engineering Southeast University Nanjing 210096 China; ^4^ LONGi Solar Technology Co. Ltd. Xi'an 710018 China; ^5^ Electronic Information Engineering College Sanjiang University Nanjing 210012 China; ^6^ International Center for Materials Nanoarchitectonics (WPI‐MANA) National Institute for Materials Science (NIMS) Namiki 1‐1 Tsukuba‐Shi Ibaraki Pref. 305‐0044 Japan; ^7^ Graduate School of Chemical Sciences and Engineering Hokkaido University Sapporo 060‐0814 Japan

**Keywords:** codoping, electrocatalysis, oxygen evolution reaction, photovoltaic water splitting, WO_2.72_

## Abstract

Oxygen evolution electrode is a crucial component of efficient photovoltaic‐water electrolysis systems. Previous work focuses mainly on the effect of electronic structure modulation on the oxygen evolution reaction (OER) performance of 3d‐transition‐metal‐based electrocatalyst. However, high‐atomic‐number W‐based compound with complex electronic structure for versatile modulation is seldom explored because of its instability in OER‐favorable alkaline solution. Here, codoping induced electronic structure modulation generates a beneficial effect of transforming the alkaline‐labile WO_2.72_ (WO) in to efficient alkaline‐solution‐stable Co and Fe codoped WO_2.72_ (Co&Fe‐WO) with porous urchin‐like structure. The codoping lowers the chemical valence of W to ensure the durability of W‐based catalyst, improves the electron‐withdrawing capability of W and O to stabilize the Co and Fe in OER‐favorable high valence state, and enriches the surface hydroxyls, which act as reactive sites. The Co&Fe‐WO shows ultralow overpotential (226 mV, *J* = 10 mA cm^−2^), low Tafel slope (33.7 mV dec^−1^), and good conductivity. This catalyst is finally applied to a photovoltaic‐water splitting system to stably produce hydrogen for 50 h at a high solar‐to‐hydrogen efficiency of 16.9%. This work highlights the impressive effect of electronic structure modulation on W‐based catalyst, and may inspire the modification of potential but unstable catalyst for solar energy conversion.

## 1 Introduction

Solar energy as the most clean and inexhaustible energy is capable of creating great value through the reasonably utilization of it.^1^ Although the advanced solar cells have realized quite efficient conversion from solar energy to electricity,^2^ energy waste is still inevitable due to the difficulty in large‐scale storage of electricity.^3^ Photovoltaic‐electrocatalytic water splitting is one of the most promising solar energy conversion methods, and provides a feasible approach to store solar energy in chemical bonds at very high efficiency,^4^ which can subtly satisfy both solar energy utilization and electricity conversion.

Highly active electrocatalyst, as well as stable operation system,^5^ is urgently needed for the photovoltaic‐electrocatalytic water splitting device.^6^ Water electrolysis simultaneously produces H_2_ and O_2_ gas, which means this reaction suffers from the buckets effect, and the short slab is exactly anodic oxygen evolution reaction (OER) as the OER has slow kinetics.^7^ We therefore focus on the study of durable and high activity OER electrocatalyst to maximize the output of hydrogen. The study on non‐noble metal catalysts, to the best of our knowledge, is seldom out of the scope of 3d metal based catalysts at present because they have significant advantages of alterable valence,^8^ appropriate oxygen binding capacity,^9^ and low cost.^10^ Various methods were adopted, including nonmetal ion doping,^11^ metal ion doping,^12^ chemical bond modulation,^13^ interface modulation,^14^ defect modulation,^15^ crystalline phase transformation,^16^ particle size modulation,^17^ and morphology design,^18^ to optimize the 3d metal based OER catalysts. By contrast, higher atomic number transition metal based OER electrocatalyst with complex electronic structure for versatile modulation is rarely developed as anodic catalyst.^19^ Zhang et al. synthesized FeCoW oxyhydroxide with unprecedentedly high performance (η = 191 mV at *J* = 10 mA cm^−2^) in 2016, and demonstrated that W can modulate 3d metal oxides to get near‐optimal adsorption energies for OER intermediates.^20^ However, few works about W based catalyst emerged over the following years. The reason is that the W oxides in its higher valence are alkaline labile,^21^ especially the WO_3_ which is even one kind of acidic oxide,^22^ but the water splitting reaction is always executed in alkaline solution because oxygen evolution is thermodynamics favorable under this pH value.^23^ This common sense induces researchers to negate the W‐based oxide catalyst, which should have potential for water oxidation in alkaline solution. Delightfully, porous WO_2_ (W is in the mixed valence of IV, V, and VI) was found to present stable OER activity by Shu et al.,^24^ inspiring us that appropriately reducing W may stabilize the oxygen evolution property of catalyst in alkaline solution.

In this study, Co and Fe ions were codoped into highly porous urchin‐like WO_2.72_ to form efficient and stable OER catalyst (Co&Fe‐WO) containing low‐valence W. The urchin‐like structure with abundant nanorods grown vertically on the surface can adequately contact with electrolyte, accelerate charge transport, and improve adsorptive separation. The codoping induced notably improved stability, enhanced conductivity, enriched surface hydroxyl groups, and enlarged electrochemical active surface area (ECSA). The reduced chemical valence of W made most sense for the persistent stability meanwhile the enriched surface hydroxyl groups and better conductivity provided the kinetic advantage for low‐overpotential OER process, which were the major points as to why the codoped catalyst showed the attractive performance. Therefore, the Co&Fe‐WO sample exhibited excellent activity (ultra‐low overpotential of 226 and 307 mV at *J* = 10 and 200 mA cm^−2^) which is, to the best of our knowledge, the top‐two performance among W‐based catalysts and exceeds most of the non‐W based catalysts. This OER catalyst as a core component of the photovoltaic‐electrocatalytic water splitting device cooperated well with the silicon solar cell to operate at high current (≈50 mA) approaching the short‐circuit current. The excellent oxygen evolution ability of Co&Fe‐WO ensured the overall water splitting system to steadily perform at a low voltage (≈1.61 V) and achieved stable hydrogen output for 50 h with an average solar to hydrogen efficiency of 16.9%.

## 2 Results and Discussion

**Figure 1:**
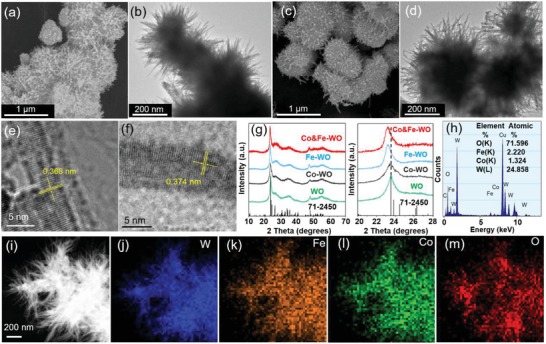
a,c) SEM images of urchin‐like WO and Co&Fe‐WO. b,d) TEM images of WO and Co&Fe‐WO. e,f) HRTEM images of WO and Co&Fe‐WO. g) XRD patterns (left) and the strongest peaks (right) of all the as‐prepared samples. h) EDX spectrum of the Co&Fe‐WO. i) HAADF‐STEM image of the Co&Fe‐WO. j–m) STEM‐EDX mapping of W, Fe, Co, and O of the Co&Fe‐WO from (i).

Classic urchin‐like WO_2.72_ (denoted as WO) was easily prepared following a hydrothermal method reported by our group.^25^ Scanning electron microscope (SEM) (**Figure** **1**a) and transmission electron microscope (TEM) (Figure 1b) images show that the as‐prepared WO sample is uniform with average diameter of about 200 nm. The doping of Co and Fe greatly changed the color of the sample from blue to yellow but brought little morphology change to the catalyst (Figure 1c,d). The SEM and TEM images of the Co‐WO and Fe‐WO catalyst are also shown in Figure S1 (Supporting Information). This special structure has the advantages of large surface area, quick electron transfer, and expedite bubble detaching ability.^26^ The Brunauer–Emmett–Teller (BET) surface area of Co&Fe‐WO is 242.7 m^2^ g^−1^, which is nearly twofold of that of WO (126.0 m^2^ g^−1^) (Figure S2, Supporting Information) although the morphologies of them are similar. Pore size distribution (Figure S3, Supporting Information) measured from BET shows that the pore size is obviously decreased after the doping (≈3.6 nm for WO, and ≈1.0 nm for Co&Fe‐WO), indicating that the metal ion doping is an effective method to form a highly porous structure for better adsorptive separation and thus the Co&Fe‐WO catalyst possesses large specific surface area. High‐resolution TEM (HRTEM) images show clear lattice fringes with the distance of 0.368 (Figure 1e) and 0.374 nm (Figure 1f) for WO and Co&Fe‐WO, respectively, indicating that the larger ions (Co ion and Fe ion) (the ion radii are listed in Figure S4, Supporting Information) are successfully introduced. The as‐prepared WO, Co‐WO, Fe‐WO, and Co&Fe‐WO were also investigated by X‐ray diffraction (XRD) measurement (Figure 1g). The WO is well‐indexed to monoclinic WO_2.72_ phase (JCPDS No. 71‐2450).^27^ The strongest diffraction peak of (010) lattice plane suggests that the growth direction of the crystalline is [010], corresponding well to the interplanar spacing (0.368 nm) measured from HRTEM image. After the ion doping, the phase of the sample is not changed, except that the main diffraction peak is shifted to smaller angle, which further confirms the result extracted from HRTEM images according to the Bragg's law.^28^ Element analysis (Figure 1h) reveals the presence of W, Co, Fe, and O, and subsequent energy dispersive X‐ray spectroscopy (EDX)‐mapping (Figure 1i–m) highlights that all the elements are homogenously distributed in the Co&Fe‐WO catalyst. Inductively coupled plasma mass spectrometry (ICP‐MS) test (Table S1, Supporting Information) was carried out after the Co&Fe‐WO catalyst was degassed because the absorbed water affected the mass analysis. The results reveal that the sample comprises of Co (1.53 wt%), Fe (3.17 wt%), and W (68.12 wt%) while the others are mostly oxygen, carbon, and the little residual water. The UV–vis–NIR absorption spectra of the samples are shown in Figure S5 (Supporting Information). A gradual redshift of the absorption edge could be observed as the following sequence: the absorption curve of WO, Co‐WO, Co&Fe‐WO, and Fe‐WO, which is in good agreement with the color changes. The single absorption band of Co&Fe‐WO further confirms the homogenous doping of Co and Fe.^29^

**Figure 2:**
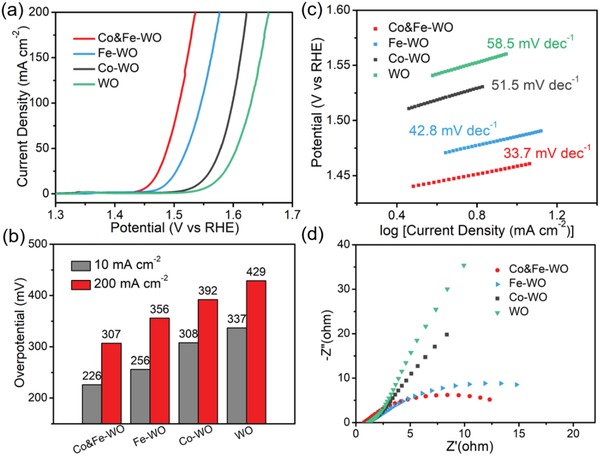
a) The OER polarization curves of samples with the scan rate of 5 mV s^−1^. b) Overpotentials for OER at the current density of 10 and 200 mA cm^−2^. c) Tafel plots and d) EIS measurement results of all the samples. All the measurements were operated in 1 m KOH solution.

A high‐performance OER catalyst is required to satisfy the following requirements: small onset potential, large ramp rate of the current density as the applied potential increase, small Tafel slope, good conductivity, and so on.^30^ Small onset potential means low OER thermodynamics. With a small potential applied between two electrodes, the electrolysis reaction could start; large ramp rate of the current density and small Tafel slope represent fast kinetics of oxygen evolution; good conductivity also promotes the electron transport and accelerates the reaction process. A series of electrochemical measurements were performed in a three‐electrode system configuration by electrochemical workstation. The electrolyte is 1 m KOH aqueous solution. **Figure** **2**a shows linear sweep voltammograms (LSVs) of anodic water oxidation by all the samples. In our electrochemical measurements, the as‐prepared WO catalyst loaded on Ni foam shows a overpotential of 337 mV (Figure 2b) at the current density of 10 mA cm^−2^ and a Tafel slope of 58.5 mV dec^−1^ (Figure 2c). Obvious improvement is observed after the Co or Fe ion was doped. Proper composition adjustment makes the optimal catalyst (Co&Fe‐WO) with the impressively low overpotential of 226 and 307 mV at the current density of 10 and 200 mA cm^−2^, respectively. This activity is, to the best of our knowledge, the second‐highest among W‐based catalyst and superior to most of the non‐W based catalysts (Table S2, Supporting Information). The Tafel slope of codoped Co&Fe‐WO (33.7 mV dec^−1^) is also notably lower than that of WO. The performances of different ratios of Co/Fe are presented in Figure S6 (Supporting Information), and the optimal Co&Fe‐WO was tested for several times (Figure S7, Supporting Information). The OER polarization curves are normalized by the BET surface area (Figure S8, Supporting Information) to confirm that the intrinsic activity of Co&Fe‐WO is notably higher than other samples. For better assessment of the OER electrode, the electrochemical performance of Co&Fe‐WO was evaluated in 2‐electrode configuration. A small onset potential of 1.48 V can be observed in the LSV curve (Figure S9, Supporting Information). Electrochemical impedance spectra (EIS) test was also introduced to study the electrochemical conductivity of the catalyst. The Nyquist plots (Figure 2d) clearly show that the incorporation of Co and Fe can pronouncedly decrease the charge transfer resistance to facilitate the OER kinetics and thus improve the oxygen evolution ability.

**Figure 3:**
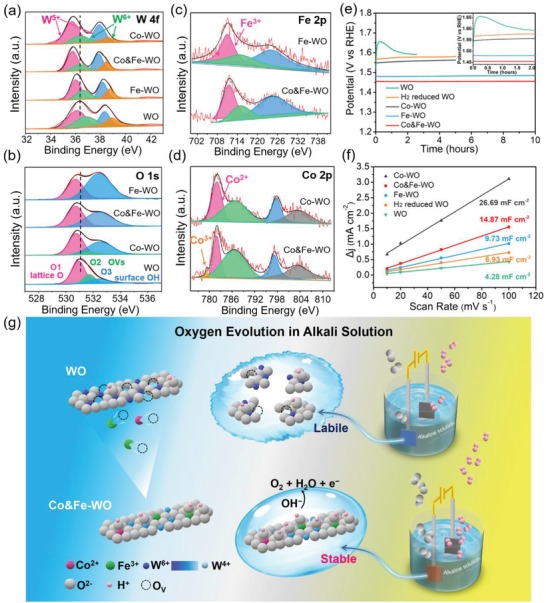
XPS data of the a) W 4f peak, b) O 1s peak, c) Fe 2p peak, and d) Co 2p peak of the samples. e) Galvanostatic measurement results for OER at the current density of 10 mA cm^−2^. f) The differences in current density (Δ*j*) at 1. 2 V versus RHE plotted against scan rate fits to a linear regression. g) Illustration of the effect of Co and Fe codoping on the activity and stability of the catalyst.

X‐ray photoelectron spectroscopy (XPS) can show the electronic structure of the catalysts,^31^ which is crucial to figure out the effect of the doped ion (Co and Fe) and how it further affects the catalytic activity. In the XPS spectrum, the peak position corresponds to the specific oxidation state of the element, and the peak area is proportional to the amount of it.^32^ The high‐resolution spectra of W 4f (**Figure** **3**a) can be deconvoluted into four peaks, corresponding to the different oxidation states of W: W^5+^ (35.7 and 37.8 eV), and W^6+^ (36.3 and 38.4 eV).^21^ The W 4f peak is evidently shifted to lower binding energy as the Co or Fe ions introduced, indicating that W^6+^ is gradually reduced to lower valence state during the doping process. The proportions of the peak area for the W^5+^ were calculated and listed in the Table S3 (Supporting Information). This value of Co&Fe‐WO (66.9%) is obviously higher than that of WO (55.3%), which further proves the decrease in the average valence of the catalyst. Judging from the W 4f spectra, the Co ion brings a stronger effect in reducing the oxidation state of W because of the more negative shift (≈0.54 eV) of the W 4f peak compared with that of the Fe‐WO (≈0.28 eV).

Figure 3b shows the O 1s XPS spectra of the catalysts. The high‐resolution spectra of O 1s can be deconvoluted into three peaks: the peak located at around 530.8 eV (O1) represents the lattice O in the WO, the peak at around 531.7 eV (O2) can be assigned to the oxygen vacancy, and the peak at higher binding energy (532.5 eV, O3) is attributed to the surface hydroxides.^15^, ^33^ After the doping of Co or Fe, the O 1s peak is shifted to lower binding energy, especially the Fe‐WO sample (shifted ≈0.33 eV). The negative shift of the binding energy means the electron accepting ability of the ion is improved. In this experiment, interestingly, both W and O shifted to lower binding energy. For the negative shift of W 4f peak, the effect of Co is more evident, while for the negative shift of O 1s peak, the effect of Fe is more evident. This negative shift makes it easier to extract electron from other elements. Therefore, we reasonably speculated that the Co and Fe ions in Co&Fe‐WO preferred to be stable in high oxidation state on the basis of the charge conservation principle. This trend is beneficial for oxygen evolution reaction, because high valence metal oxide can work as the active sites.^17^, ^34^ We next proceeded to analyze the electronic states of Fe and Co determined from the 2p region of high resolution XPS spectra. As Figure 3c shown, the peaks (located at 711.5 and 724.7 eV) in Fe 2p spectra for Fe‐WO and Co&Fe‐WO are assigned to high valence Fe^3+^ which is generally OER favorable.^35^ The Co 2p peaks (located at 781.8 and 797.7 eV) are mainly attributed to Co^2+^,^36^ and a little amount of Co^3+^ (the binding energy is 780.1 eV) is observed in Co&Fe‐WO sample (Figure 3d).^34^ The UV–vis–NIR spectrum of the WO (Figure S5, Supporting Information) shows a large absorption tail at the range of 450 to 840 nm, which presents the oxygen vacancies (OVs) in oxides.^37^ However, the intensity of this tail decreases evidently after the doping of Co, and even disappears after the doping of Fe. This result is well consistent with the change of the peak of OVs (531.7 eV) after the doping in O XPS spectra (Figure 3b). In the field of defect chemistry, low valence metal ion is always accompanied with the oxygen vacancy.^38^ We inferred that the incorporation of Co and Fe ions highly consumed the oxygen vacancy, thereby promoted the stabilization of them at high valence state.

The stability tests were performed by chronopotentiometry at the current density of 10 mA cm^−2^. The WO catalyst presents very poor stability in contrast with the Co&Fe‐WO catalyst, which presents extreme durability during the test lasted for 10 h (Figure 3e). Almost no change occurred on the potential applied on Co&Fe‐WO electrode. The *I*–*t* curve (Figure S10, Supporting Information) of the optimal sample was also measured, and the current density is still very stable. The initial current density is 12.05 mA cm^−2^, and maintained at 11.92 mA cm^−2^ after testing for 10 h. To the best of our knowledge, the alkaline resisting WO_2_ has stable oxygen evolution ability. We therefore turned instead to investigate whether the valence change accounts for the stabilization of the catalyst. On reducing the valence state of W, the WO sample was reduced by the H_2_ for 2 h and characterized by XRD measurement (Figure S11, Supporting Information). The emerging planes of (231), (232), and (113), as well as the shoulder peak (at 23.32°) next to the strongest peak correspond well to the WO_2_ (JCPDS No. 82‐0728). The color of the sample became darker, indicating the valence of W decreased (the color of W oxide is darkened as the valence decreased).^24^, ^39^ The dark color was induced by large amount of oxygen vacancies, which was manifested as a broad light absorption in the UV–vis–NIR spectrum (Figure S12, Supporting Information). The reduced sample was tested in the same configuration as that of other samples, and the stability was pronouncedly improved after the reduction process. Furthermore, the WO and Co&Fe‐WO were oxidized in air and tested. It is found that the high oxidation W oxide electrodes were not durable (Figures S13 and S14, Supporting Information), which demonstrates that the reduction of the valence of W is the main reason for the enhancement of activity durability. However, the H_2_ reduced WO and the most thoroughly reduced Co‐WO (the most negative shift in W 4f XPS spectrum) catalysts do not perform the best stability, a slight increase in the potential happened during the tests. We speculated that the decrease in the oxidation state of W is not the only reason for the improvement in stability.

As shown in Figure 3b, the proportion of the O3 became larger as the content of Fe increased. The corresponding ratio of O3/(O1 + O2) was calculated and listed in Table S4 (Supporting Information), which can quantitatively reveal the change of the area of O3 peak. The ratios of O3/(O1 + O2) are 0.2152, 0.9021, 1.2075, and 1.6580 for the WO, Co‐WO, Co&Fe‐WO, and Fe‐WO, respectively. The increased amount of OH group carries tangible improvement of OER activity according to one widely held view that hydroxide can act as the oxygen evolution site,^40^ and the increase in O3 peak also means that the hydrophilicity of the catalyst is promoted to form a more aerophobic surface. The porous urchin‐like catalyst has abundant nanorods grown vertically on the surface. This structure can faster the charge transport along the surface and hence accelerate the water oxidation reaction. With the hydrophilic surface and the highly porous structure, once the oxygen is formed, the bubble can quickly release from the surface of the catalyst^41^ and thus prevent the oxidation of WO base. Therefore, the codoping not only introduced high reactivity of active sites to boost OER, but also contributed to the stabilization of electrocatalyst.

We also investigated the electrochemical active surface area to further elucidate the origin of the superior performance of the Co&Fe‐WO. Normally, an increase of ECSA provides additional active area and leads to the enhancement of catalytic activity.^42^ The value of ECSA could be deduced from the electrochemical double‐layer capacitance (*C*_dl_)^43^ measured by cyclic voltammetry (CV). Here, the measurements of cyclic voltammetry (Figure S15, Supporting Information) were carried out at various rates in the operating potential range of 1.15 to 1.25 V versus reversible hydrogen electrode (vs RHE). The *C*_dl_ values were obtained by calculating the slope of the lines in Figure 3f. Both the Co and Fe obviously increase the ECSA of the catalyst. The Co&Fe‐WO possesses a high active surface area (14.87 mF cm^−2^) which is 3.47 times as large as that of WO (4.28 mF cm^−2^). The *C*_dl_s of the Co‐WO and the Fe‐WO are 26.69 and 9.73 mF cm^−2^, respectively. The sample with the highest Co content possesses the largest active area, but does not show the best OER activity, which may be ascribed to that the insufficient binding capacity to OH and large charge transfer resistance weaken the benefit brought by the high active surface. Subsequently, the polarization curves (Figure S16, Supporting Information) normalized by the ECSA show that the Co&Fe‐WO still presents the best performance of oxygen evolution, manifesting that the enhancement in active area was not the exclusive factor for promoting the activity.

For the constructing of W‐oxide‐based OER electrocatalyst here, the method of ion doping induced many positive effects (Figure 3g): 1) reduced Tafel slope, 2) promoted stability, 3) improved conductivity, 4) increased amount of surface hydroxyl groups, and 5) enlarged ECSA. The codoping of Co and Fe is crucial because the codoped catalyst shows the lowest Tafel slope, best stability and conductivity. The excellent stability in alkaline solution is mainly attributed to the decrease in the valence of W, and the enriched surface hydroxy also works. In terms of the ability to catalyze OER, Co and Fe codoped catalyst has the highly reactive sites, the promoted conductivity and the porous urchin‐like structure with larger surface area and good separation property for adsorptive, thus presenting ultra‐low overpotential and good polarization property.

**Figure 4:**
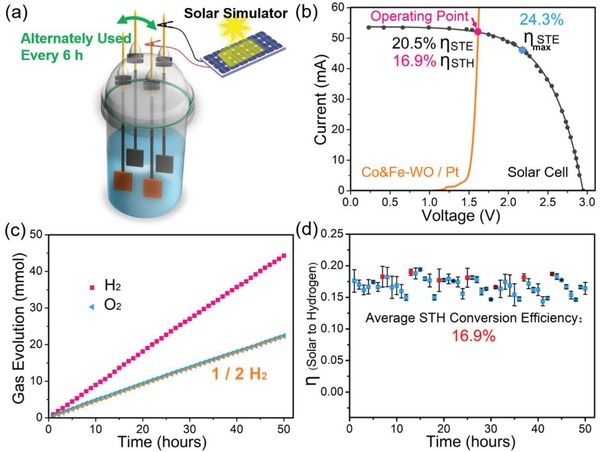
a) The device for photovoltaic‐electrocatalytic water splitting. b) *J*–*V* curves of the silicon solar cell under 0.87‐fold AM 1.5 (87.13 mW cm^−2^) illumination provided by a solar simulator, and the Co&Fe‐WO / Pt electrodes in a two‐electrode configuration; the η_STE_ is solar to electricity conversion efficiency, and the η_STH_ represents solar to hydrogen conversion efficiency. c) The average yields of H_2_ and O_2_ for the two pairs of electrodes, the orange line represents the half yield of the H_2_. d) Solar to hydrogen conversion efficiency of the PV‐electrocatalytic water splitting device for every hour during the measurement of 50 h. The red point represents the efficiency after the naturally discharging.

Considering the efficient utilization of solar energy and the difficulties in large‐scale storage of electricity, we constructed a photovoltaic‐electrocatalytic water splitting device (**Figure** **4**a) to achieve two successive steps: first, converting the solar energy to electricity, and then converting the electricity to the sustainable hydrogen fuels. A piece of Ni foam sputtered with Pt was used as the cathode (catalyst loading is about 1.1 mg cm^−2^), and the Co&Fe‐WO was coated on Ni foam (1.0 mg cm^−2^) as the oxygen evolution anode. The water splitting reactor was connected to a closed gas circulation system equipped with online gas chromatography. External silicon film solar cell was illuminated by a solar simulator (the light spectrum is shown in Figure S17, Supporting Information) to accomplish the energy input. The light power in the range of 0.70 to 1.24‐fold AM 1.5 was supplied to the device, and the H_2_ yield (Figure S18a, Supporting Information) is nearly proportional to the light power under the relative weak light illumination. The solar‐to‐hydrogen efficiency was decreased while stronger light (≥1.12‐fold AM 1.5) was input (Figure S18b, Supporting Information). Here, the illumination power of 0.87‐fold AM 1.5 was selected in subsequent tests, and the maximum solar photovoltaic conversion efficiency under this power is 24.3%. The operating voltage of about 1.61 V was determined by the *J*–*V* curves of the solar cell and the electrodes (Figure 4b), because the current (≈51 mA) under this voltage is very close to the short‐circuit current to realize the high output of H_2_. Larger voltage leads to more energy loss and smaller current although the corresponding solar‐to‐electricity conversion efficiency is higher (Figure S19, Supporting Information). As a contrast, LSV curve of a pair of Pt sputtered Ni foam electrodes was tested in the same configuration (Figure S20, Supporting Information), which shows that larger voltage is necessary for Pt/Pt electrodes to reach the same current, revealing that the as‐prepared Co&Fe‐WO/Pt electrodes are more energy‐efficient. Remarkably, a phenomenon occurred during the tests of Co&Fe‐WO/Pt electrodes that the voltage between two electrodes gradually increased due to the effect induced by the double‐layer capacitance. Surface charge was constantly accumulated, accompanied by the decreasing of the actual voltage for water splitting. Therefore, two pairs of electrodes were alternately used every 6 h in this experiment. The electrodes could be naturally discharged when not in use so that the activity of them got back close to the optimum value when used again. In this way, the stability test of 100 h was performed and both of the two pairs worked for 50 h. The average yields of H_2_ and O_2_ are shown in Figure 4c, and the gas evolution rate of H_2_ (885.8 µmol h^−1^) is nearly twofold of that of O_2_ (447.5 µmol h^−1^) with the initial Faradic efficiency of 96.8%. Stable solar to hydrogen (STH) conversion was achieved under the low voltage (≈1.61 V) and high current value (≈51 mA), and finally the average STH conversion efficiency is 16.9% (Figure 4d).

## 3 Conclusion

In summary, Co and Fe ions were homogenously codoped in the porous urchin‐like WO_2.72_ to create a new alkaline‐available OER electrocatalyst (Co&Fe‐WO) which was applied to the PV‐water splitting device. The 3d metals exerted an electron interacting with W to lower the valence state of W, which effectively modified the alkaline‐labile WO_2.72_ to be alkaline solution available. The codoping enabled Co and Fe ions to stabilize in high valence state and planted abundant surface hydroxides to not only act as reactive sites but also protect the catalyst base. The Co&Fe‐WO sample shows very low overpotential of 226 mV (*J* = 10 mA cm^−2^), small Tafel slope of 33.7 mV dec^−1^ and good conductivity. The PV‐water electrolysis device realized stable solar‐to‐hydrogen conversion for 50 h with the average η_STH_ of 16.9%. This work propelled the exploration of W‐based OER electrocatalyst, which has complex electronic structure for versatile modulation and paved an avenue to effectively improve the stability of catalyst for efficient energy conversion applications.

## 4 Experimental Section

*Preparation of Urchin‐Like Co&Fe‐WO, Co‐WO, Fe‐WO, and WO*: Anhydrous WCl_6_ (1.2 g), CoCl_2_ (0.3 g), and FeCl_3_ (0.3 g) were dissolved in 60 mL absolute ethanol and stirred for 20 min to form a homogeneous solution. The solution was then moved into a 100 mL Teflon‐lined stainless steel autoclave and held at 180 °C for 20 h. A yellow powder (Co and Fe codoped WO_2.72_) was obtained and finally washed with ethanol and distilled water alternately. This product was denoted as Co&Fe‐WO.

The contrast samples, Co doped WO_2.72_ (Co‐WO), Fe doped WO_2.72_ (Fe‐WO), and WO_2.72_ (WO) were synthesized following the similar method, except without FeCl_3_, CoCl_2_ and either of them, respectively.

*Characterizations*: X‐ray diffraction patterns were recorded by X‐ray diffractometer (D8 Advanced, Bruker, Germany). The morphologies of the samples were observed by the transmission electron microscope (Tecnai G2 F20, FEI, America) and scanning electron microscope (Nanosem 430, FEI, America). Chemical valence analysis was performed by X‐ray photoelectron spectroscopy (Escalab 250Xi, Thermo Scientific, America). Elemental composition of the catalysts was detected by ICP‐MS (Optima 5300DV, Perkin Elmer, America). The Brunauer–Emmett–Teller area was measured by nitrogen physisorption (Autosorb‐iQ2, Quantachrome, America). The UV–vis–NIR absorption spectra of the samples were recorded via a UV–visible–NIR spectrophotometer (UV‐2700, Shimadzu, Japan).

*Electrochemical Measurements*: 5 mg of catalyst powder was dispersed in a mixture of water (100 µL) and ethanol (300 µL), and then 20 µL of Nafion solution (5 wt% in water) was added to this suspension. After ultrasonic stirred for 30 min, 80 µL of the homogeneous ink was deposited onto a 1 × 1 cm^2^ Ni foam substrate (catalyst loading, 1.0 mg cm^−2^). The Ni foam was pretreated with diluted H_2_SO_4_ solution and washed by distilled water until the pH of water reached neutral.

Electrochemical performance was evaluated in a three‐electrode configuration by electrochemical workstation (CHI660E, CHI instrument). All the measurements were carried out in 1.0 m KOH aqueous solution. The reference electrode was a Ag/AgCl (3 m KCl) electrode, and the counter electrode was a Pt wire electrode. The linear sweep voltammetry test was performed at a scan rate of 5 mV s^−1^. All the LSV curves are iR corrected by compensating the series resistance (*R*_s_) values which were extracted from electrochemical impedance Spectroscopy. Double‐layer capacitance (*C*_dl_) values were deduced from cyclic voltammetry measurements which were cycled between 1.15 to 1.25 V (vs RHE) at different scan rates. The electrochemical impedance measurement was evaluated at 1.45 V (vs RHE) from 1 × 10^6^ to 0.1 Hz in the same configuration as LSV measurements.

*Photovoltaics‐Water Splitting Evaluation*: The PV‐water splitting reaction was performed in a water splitting reactor which was connected to a closed gas‐circulation system (OLPCRS‐3, Shanghai Boyi Scientific Instrument Co., China). The electrolysis reaction was performed in 1 m KOH solution with the distance of 1 cm between the anode (Co&Fe‐WO/NF) and cathode (Pt coated Ni foam). A solar simulator (XES‐50S1‐RY, San‐Ei Electric Co., Japan) was used as the light source to input the solar energy to a crystalline silicon solar cell. The illumination area of the silicon film solar cell is 4.73 cm^2^. The H_2_ and O_2_ evolution was detected by online gas chromatography equipped with thermal conductivity detector (GC‐2014C, Shimadzu Corp., Japan).

*Calculation of Solar‐To‐Hydrogen Energy Conversion Efficiency*: The Solar‐to‐Hydrogen conversion efficiency for every hour was calculated by the following two methods:

one is on the basis of the standard molar enthalpy of combustion (−285 kJ mol^−1^)

(1) $$\eta_{1}\;\;=\;\;\frac{\mathrm{standard}\;\mathrm{molar}\;\mathrm{enthalpy}\;\mathrm{of}\;\mathrm{combustion}\;\left(\mathrm{kJ}\;\;{\mathrm{mol}}^{-1}\right)\;\;\times \;\;H_{2}\;\mathrm{moles}\;\left(\mathrm{mol}\right)}{\mathrm{illumination}\;\;\mathrm{power}\;\left(W\right)\;\;\times \;\;\mathrm{time}\left(s\right)}$$

where the illumination powder was measured by an optical power meter (PM 100D, Thorlabs, America), the range of measurement is 190–20 000 nm, the energy of H_2_ was obtained according to the standard molar enthalpy of combustion (−285 kJ mol^−1^). For the calculation of every hour, the H_2_ moles represent the H_2_ evolution amount in 1 h, and the time equals to 3600 s.

The other is according to the thermodynamic potential (1.23 V) of water splitting, and assuming a 100% Faradaic efficiency

(2) $$\eta_{2}\;\;=\;\;\frac{J\left(A\right)\;\;\times \;\;1.\mathrm{23}\;\;V\;\;\times \;\;\mathrm{100}\%}{\mathrm{illumination}\;\;\mathrm{power}\;\;\left(W\right)}$$

where *J* is the current for operating, the value of illumination powder is the same with the above‐mentioned calculation.

The example calculations are as following:the average amount of H_2_ per hour is 0.8858 mmol,

η_1_ = 285 kJ mol^−1^ × 0.8858 mmol/ (0.412 W × 3600 s) = 16.9%

η_2_ = 51.4 mA × 1.23 V × 100%/ 0.412 W = 15.3%

*Calculation of Faradaic Efficiency*: Faraday efficiency is defined as the ratio of the actual yield to the theoretical yield. The calculation is as following

(3) $$\mathrm{Faraday}\;\mathrm{Efficiency}\;\;=\;\;\frac{m\left(\mathrm{mol}\right)\;\;\times \;\;n\;\;\times \;\;F\left(C\;\;{\mathrm{mol}}^{-1}\right)}{J\left(A\right)\;\;\times \;\;\mathrm{time}\left(s\right)}\;\;\times \;\;100\%$$

where *m* is the actual moles of the product, *n* is the number of electron transfer, *F* is the Faraday constant (96 485 C mol^−1^), *J* is the current value obtained from the linear sweep voltammetry in the same configuration as PV‐electrocatalytic water splitting, and *t* is the reaction time.

An example calculation is given as follows: the current for H_2_ evolution is about 51.4 mA, the amount of detected H_2_ for the first hour is 0.928 mmol, *n* equals to 2 and time equals to 3600 s. The Faraday Efficiency is calculated as: 0.928 mmol × 2 × 96 485 C mol^−1^/ (51.4 mA × 3600 s) × 100% = 96.8%

## Conflict of Interest

The authors declare no conflict of interest.

## Supporting information

Supplementary
