# Supplementary material for: Co and Fe Codoped WO2.72 as Alkaline‐Solution‐Available Oxygen Evolution Reaction Catalyst to Construct Photovoltaic Water Splitting System with Solar‐To‐Hydrogen Efficiency of 16.9%
Source: Adv Sci (Weinh). 2019 Jul 11;6(16):1900465. doi: 10.1002/advs.201900465 (PMC6702762; doi:10.1002/advs.201900465)
Supplement: Supplementary file 1 — Supplementary [file ADVS-6-1900465-s001.pdf]

Copyright WILEY-VCH Verlag GmbH & Co. KGaA, 69469 Weinheim, Germany, 2019.

ADVANCED SCIENCE
Open Access

Supporting Information

for *Adv. Sci.,* DOI: 10.1002/advs.201900465

Co and Fe Codoped $WO_{2.72}$ as Alkaline-Solution-Available Oxygen Evolution Reaction Catalyst to Construct Photovoltaic Water Splitting System with Solar-To-Hydrogen Efficiency of 16.9%

Huayu Chen, Lizhu Song, Shuxin Ouyang, Jianbo Wang, Jun Lv,* and Jinhua Ye**

Supporting Information

Co and Fe Codoped $WO_{2.72}$ as Alkaline-Solution-Available Oxygen Evolution Reaction Catalyst to Construct Photovoltaic Water Splitting System with Solar-To-Hydrogen Efficiency of 16.9%

*Huayu Chen, Lizhu Song, Shuxin Ouyang,*Jianbo Wang, Jun Lv,* and Jinhua Ye**

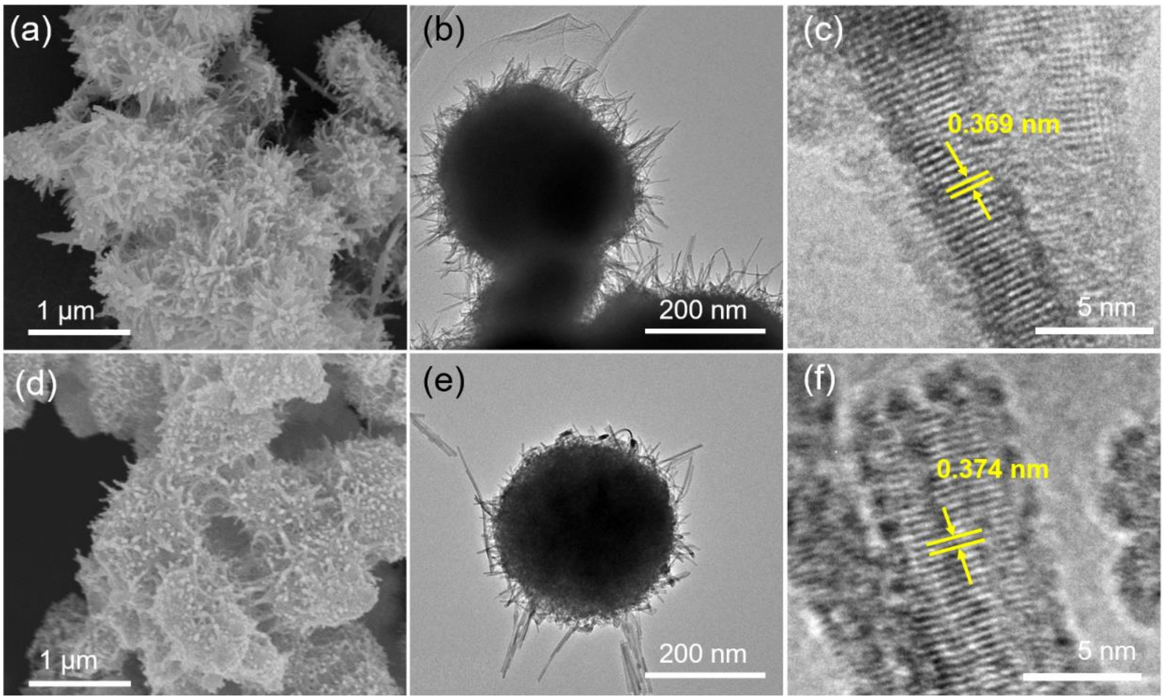

Figure S1. a-c) SEM image, TEM image and HRTEM image of urchin-like Co-WO. d-f) SEM image, TEM image and HRTEM image of the Fe-WO.

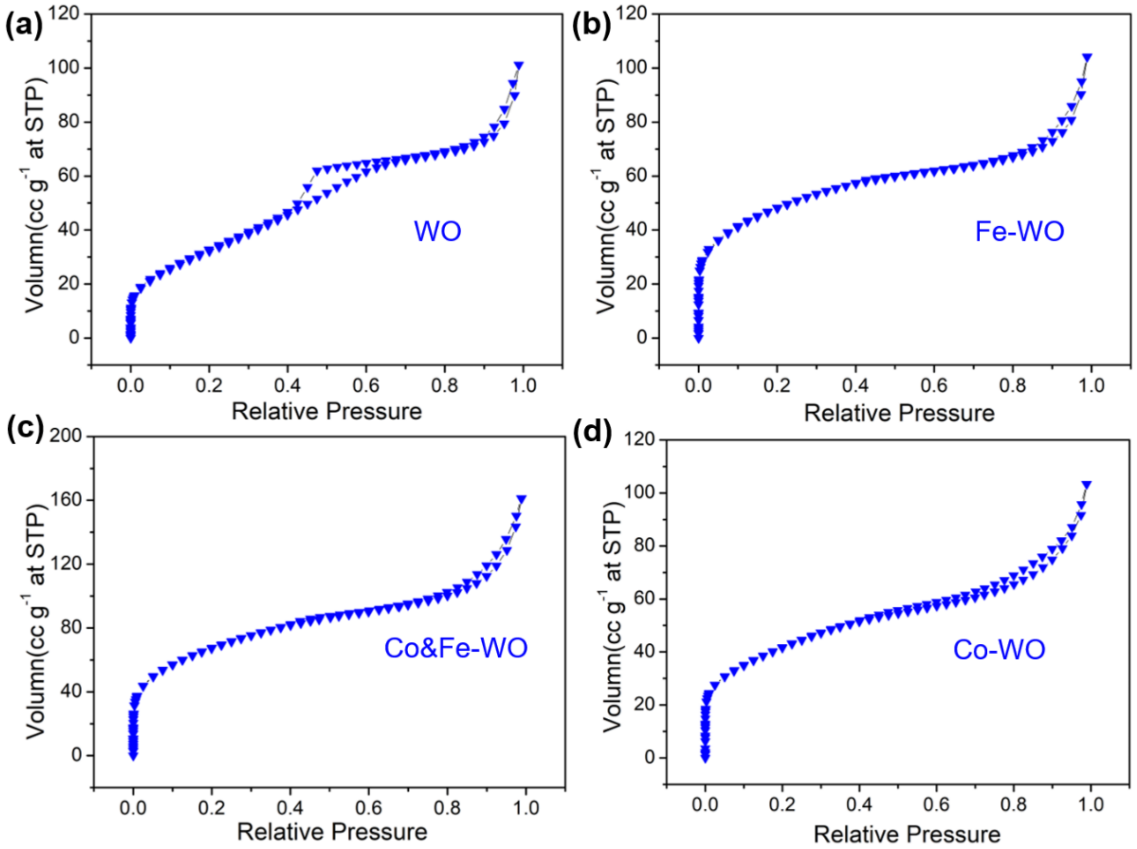

Figure S2. N_2 adsorption/desorption isotherms of a) WO, b) Fe-WO, c) Co&Fe-WO and d) Co-WO.

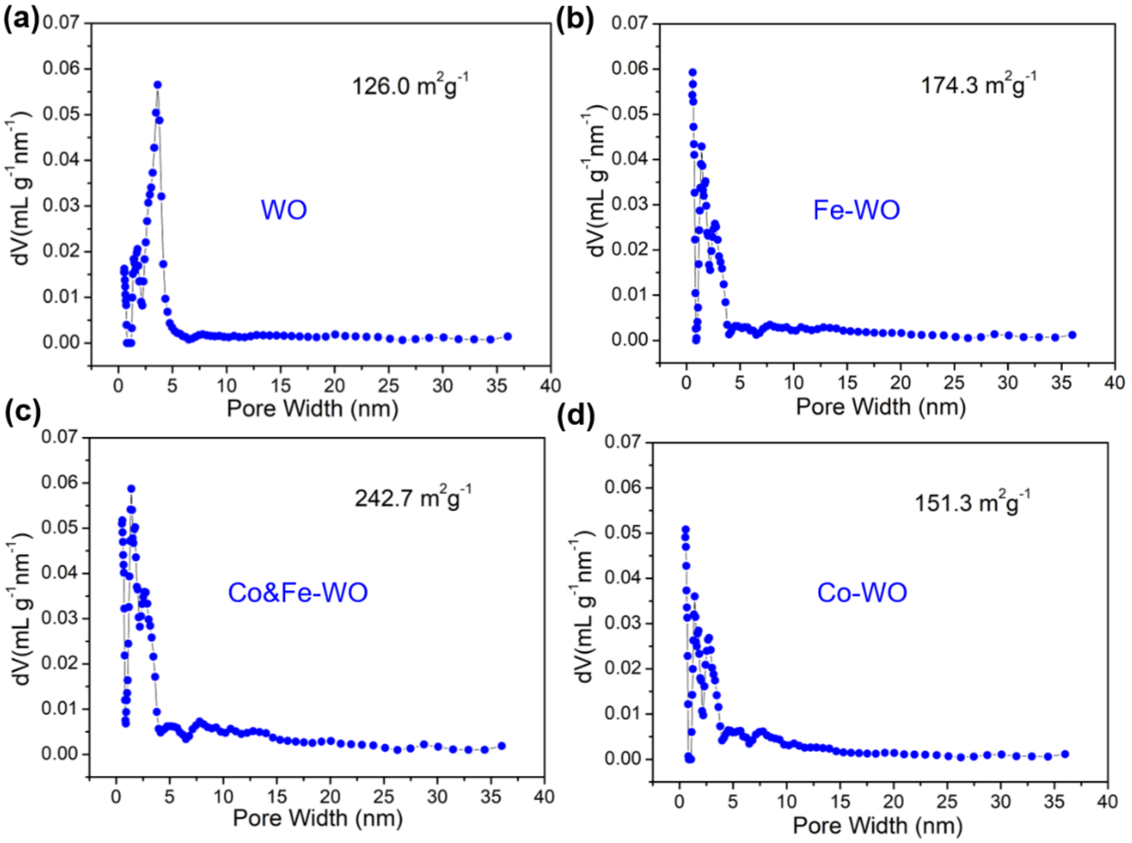

Figure S3. Pore size distribution of a) WO, b) Fe-WO, c) Co&Fe-WO and d) Co-WO, the corresponding pore size are listed in the picture.

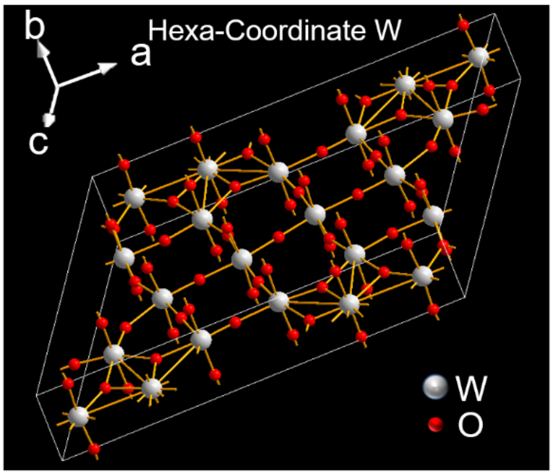

Hexa-coordinate metal ion	Effective ionic radius (pm)#
Co^{2+}	65.0 LS<br>74.5 HS
Fe^{3+}	55.0 LS<br>64.5 HS
W^{6+}<br>W^{5+}	60.0<br>62.0

Referred to Lange's Handbook of Chemistry

Figure S4. Diagram of the unit cell structure for $WO_{2.72}$ and the effective ionic radius of Co^{2+}, Fe^{3+}, W^{5+} and W^{6+} referred to Lange's Handbook of Chemistry.

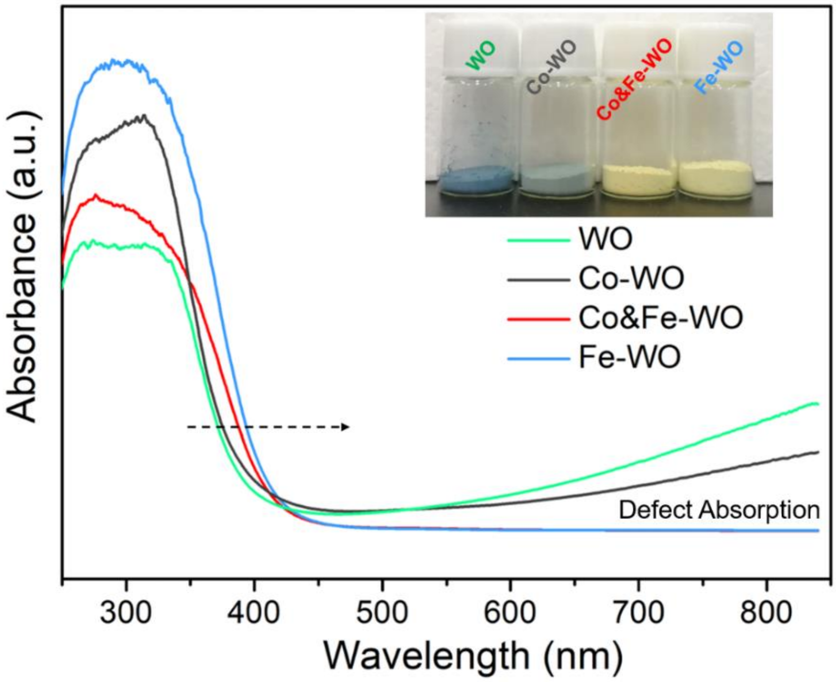

Figure S5. UV-vis-NIR spectra and photographs of all the samples.

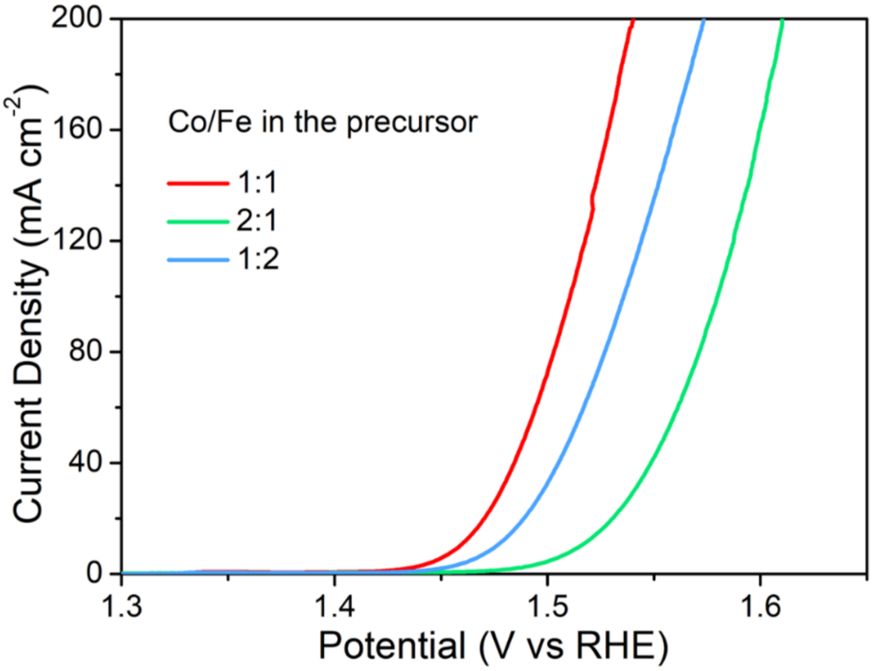

Figure S6. The OER polarization curves of the samples with different Co/Fe ratio in the precursor, the scan rate is 5 mV s^{-1}.

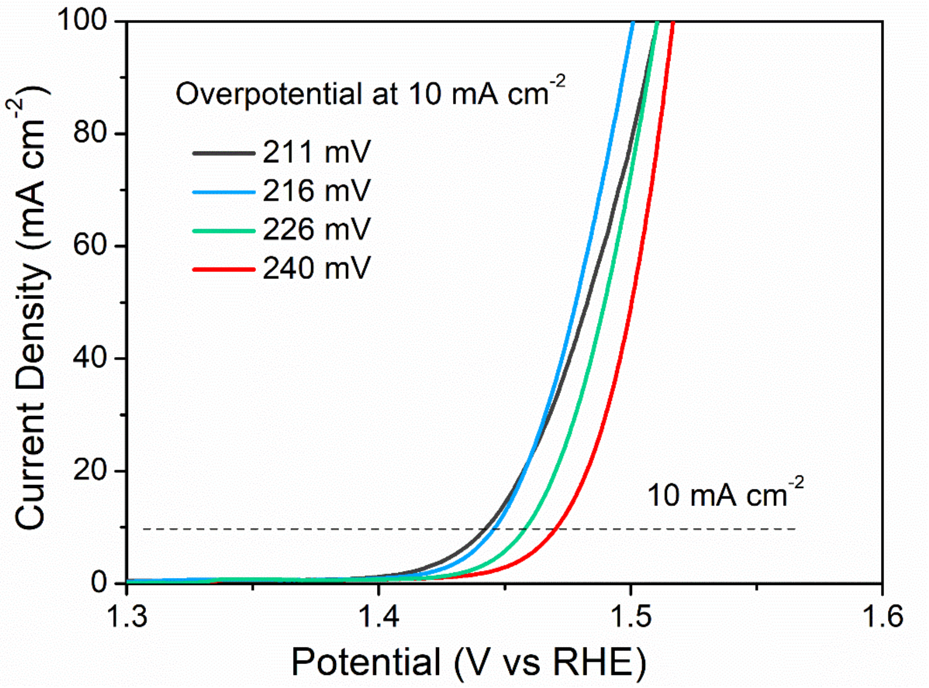

Figure S7. The OER polarization curves of the optimal sample (Co&Fe-WO) for different measurements with scan rate of 5 mV s^{-1}.

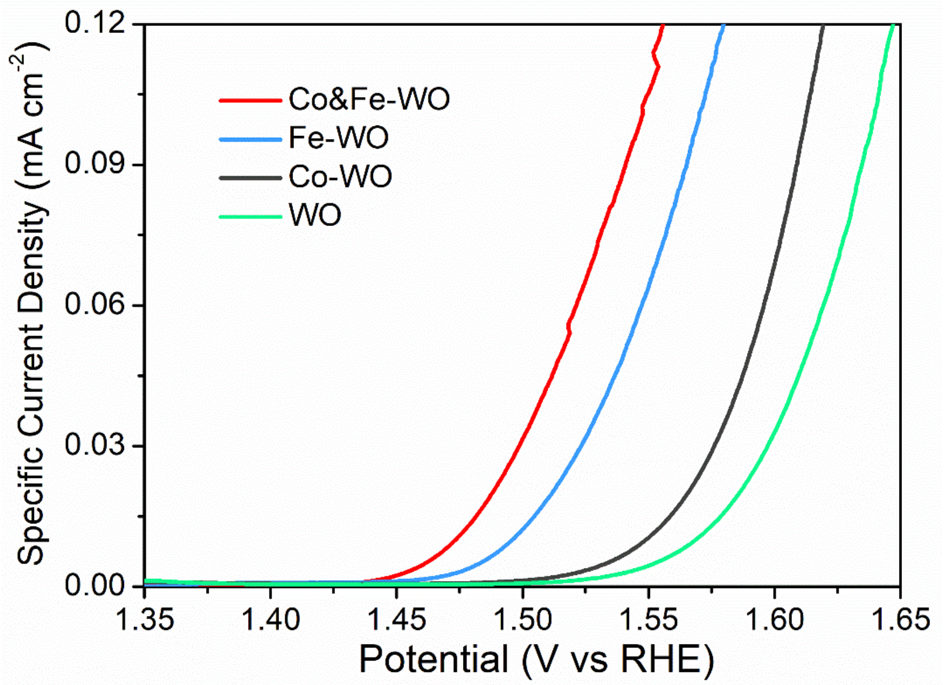

Figure S8. The OER polarization curves normalized by the BET surface area for all the catalysts with scan rate of 5 mV s^{-1}.

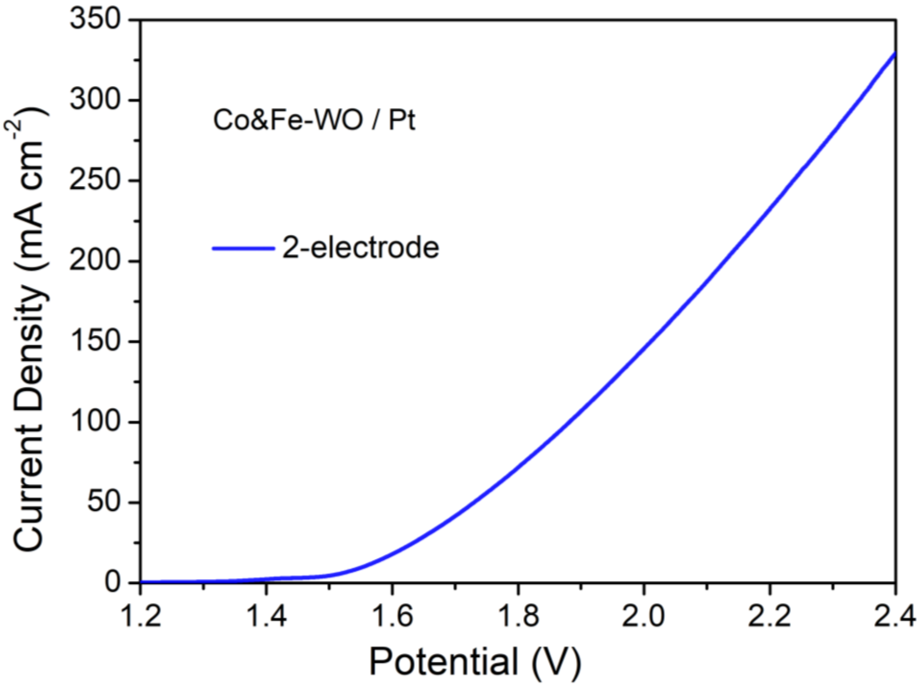

Figure S9. The LSV curve for the Co&Fe-WO/Pt mesh electrode in a 2-electrode configuration with scan rate of 5 mV s^{-1}.

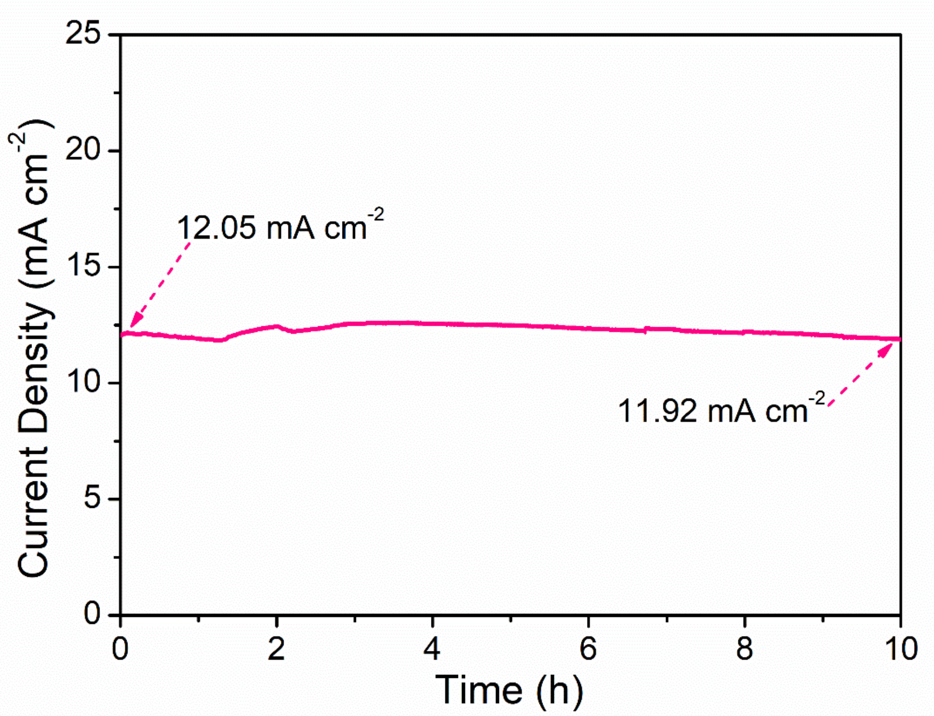

Figure S10. The I-t stability curves of the optimal sample (Co&Fe-WO).

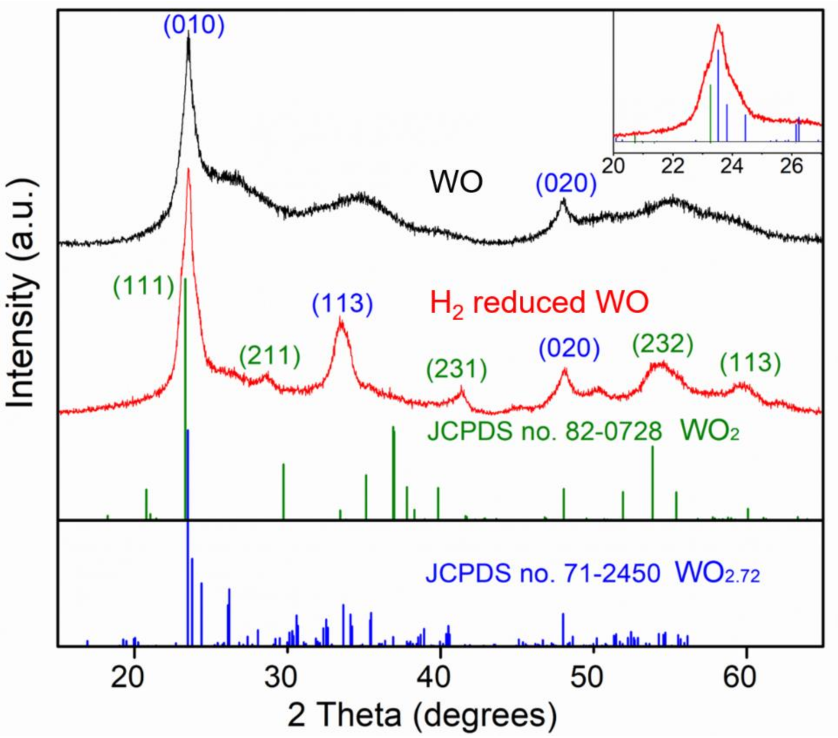

Figure S11. XRD patterns of the H_2 reduced WO catalyst.

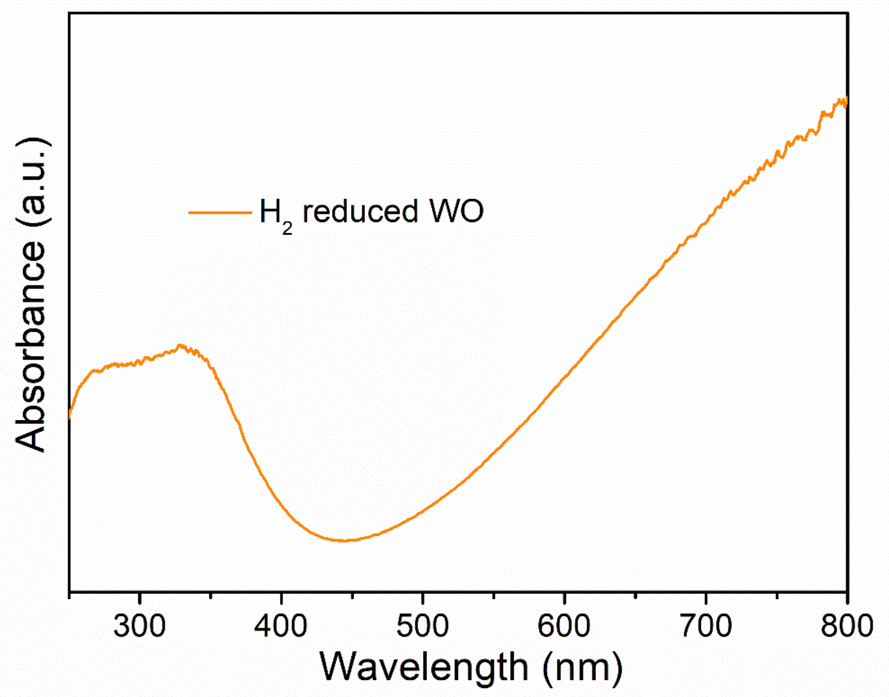

Figure S12. UV-vis-NIR spectrum of H_2 reduced WO.

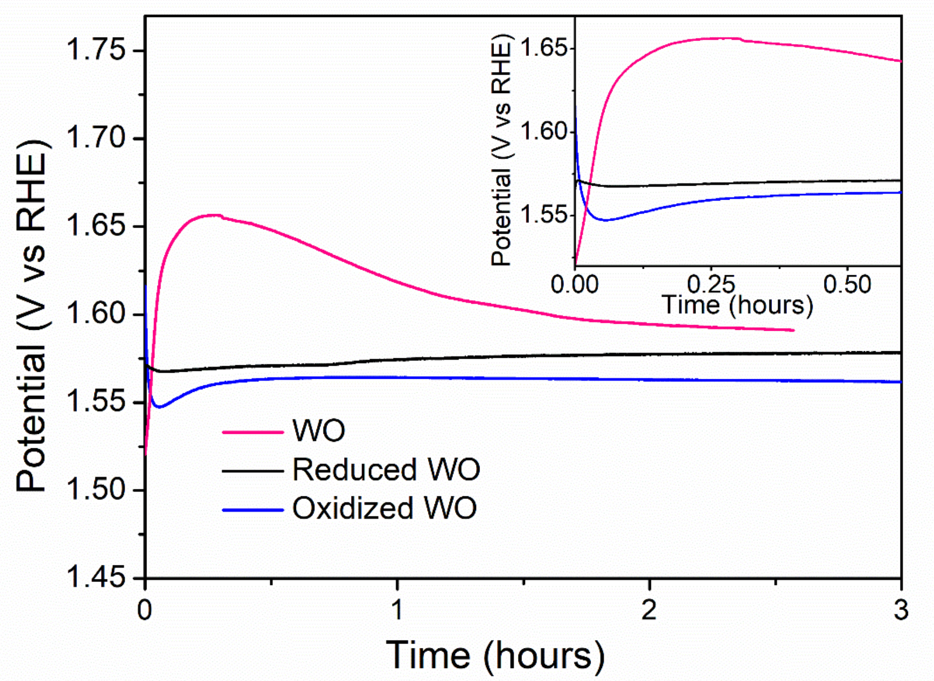

Figure S13. Galvanostatic measurement results for the WO, reduced WO and oxidized WO at the current density of 10 mA cm^{-2}.

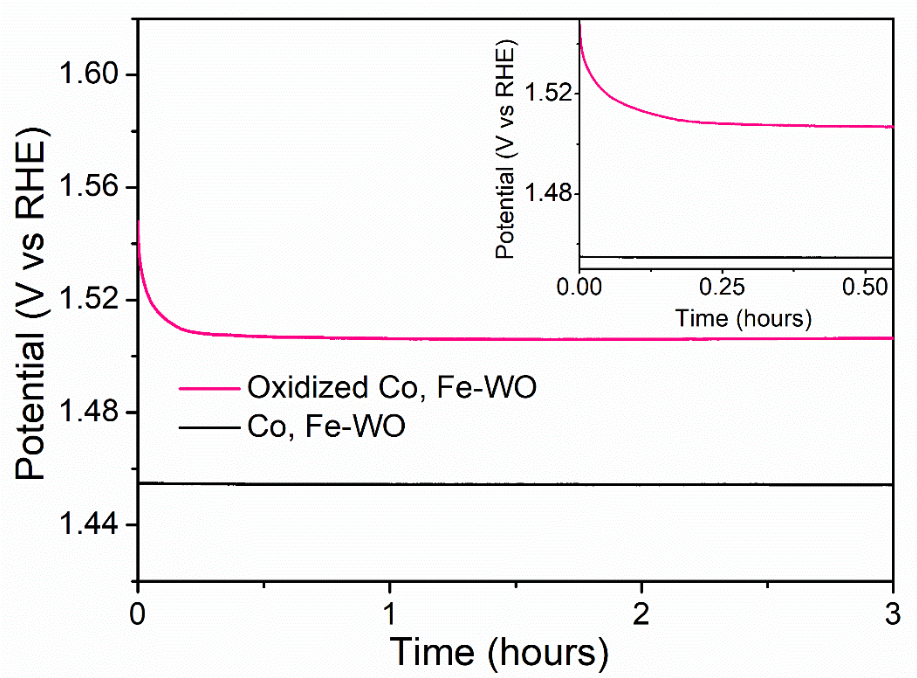

Figure S14. Galvanostatic measurement results for the Co&Fe-WO and reduced Co&Fe-WO at the current density of 10 mA cm^{-2}.

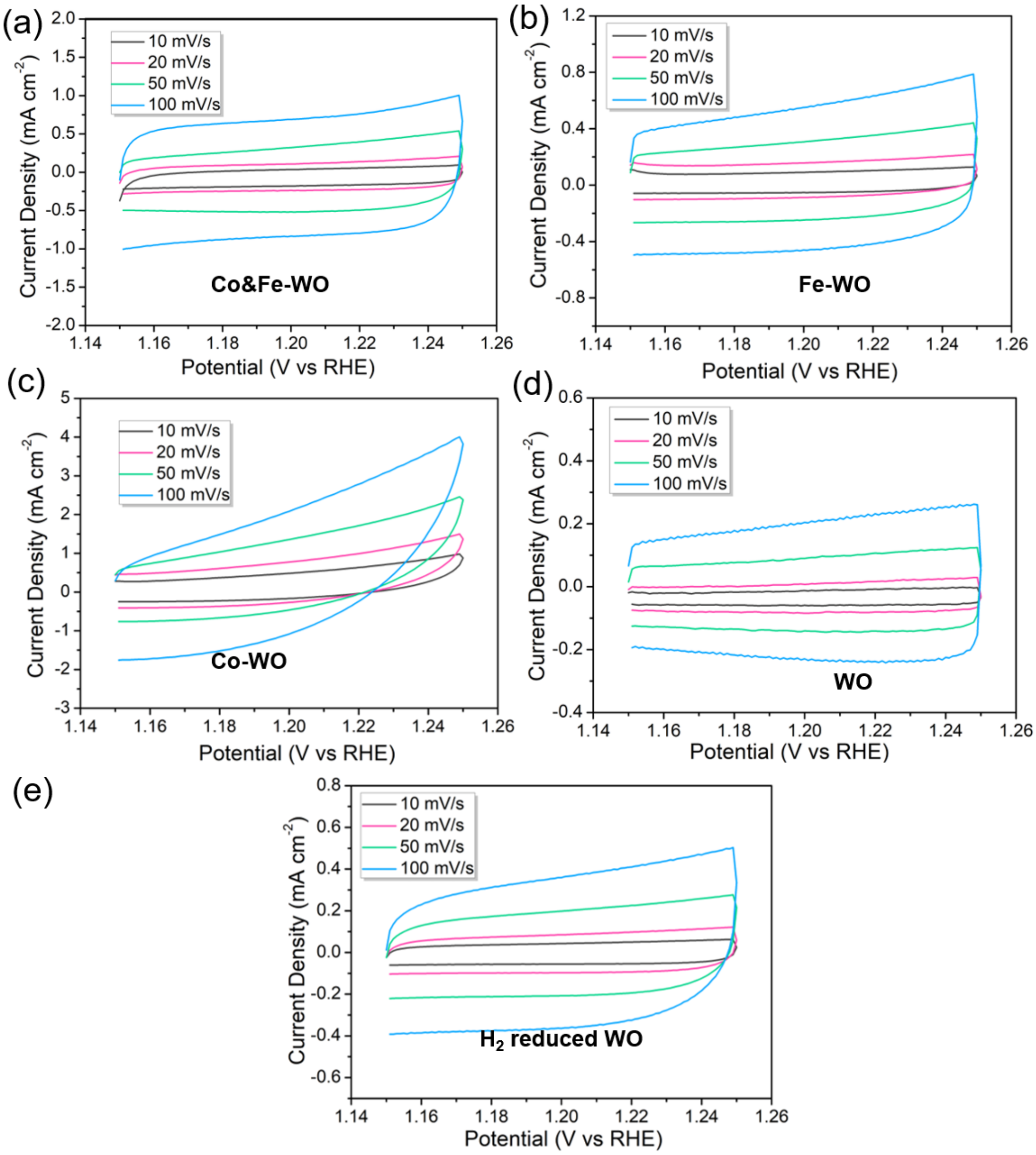

Figure S15. The cyclic voltammetry curves cycled in the range of 1.15 to 1.25V vs RHE for all the samples at different scan rates.

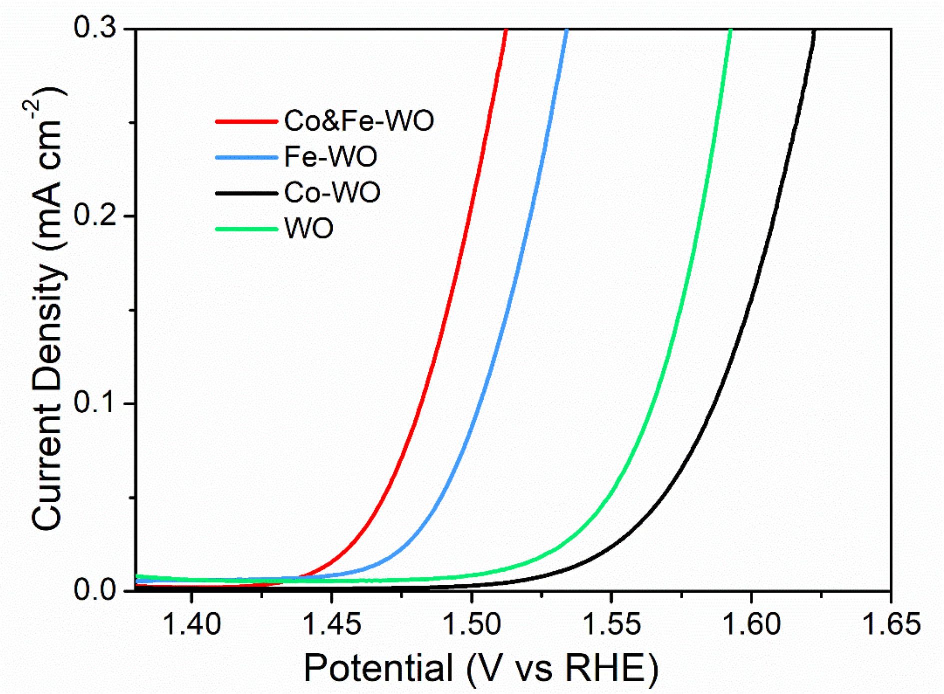

Figure S16. The OER polarization curves of all the samples in 3-electrode configuration in 1 M KOH aqueous electrolyte with scan rate 5 mV s^{-1}. (Normalized by ECSA)

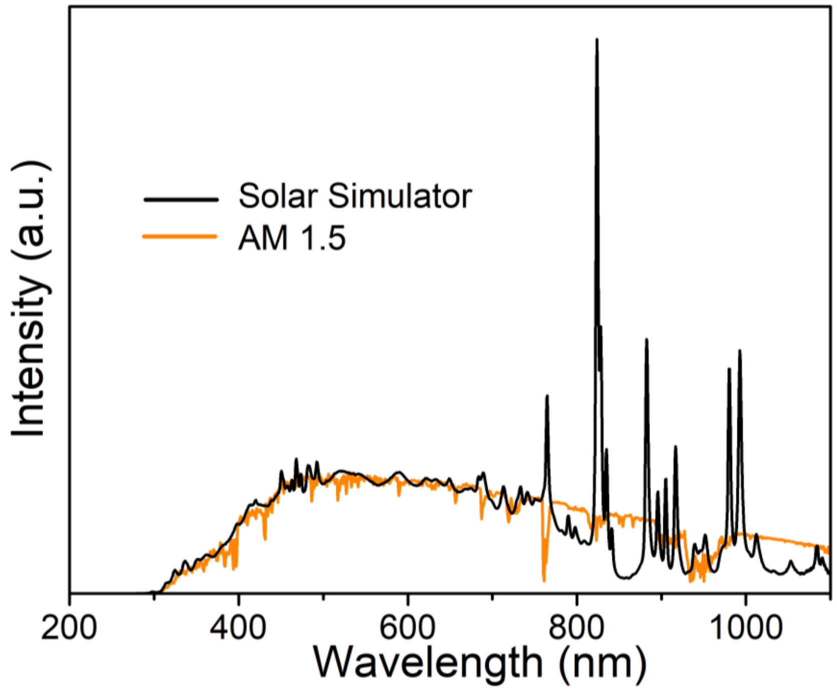

Figure S17. The spectrum of the solar simulator as the input light source for PV-electrocatalytic water splitting device and the standard solar spectrum.

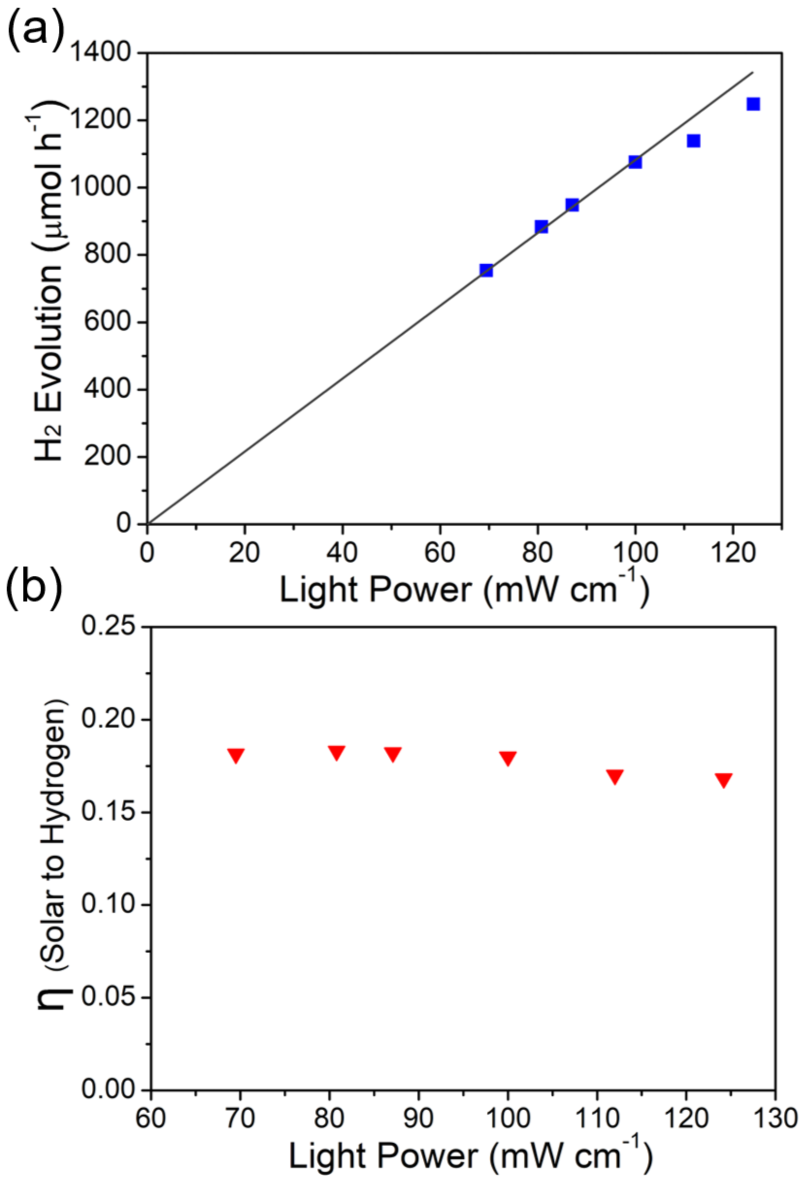

Figure S18. a) H_2 evolution, and b) the solar to hydrogen conversion efficiency of the device under the illumination of different light powers.

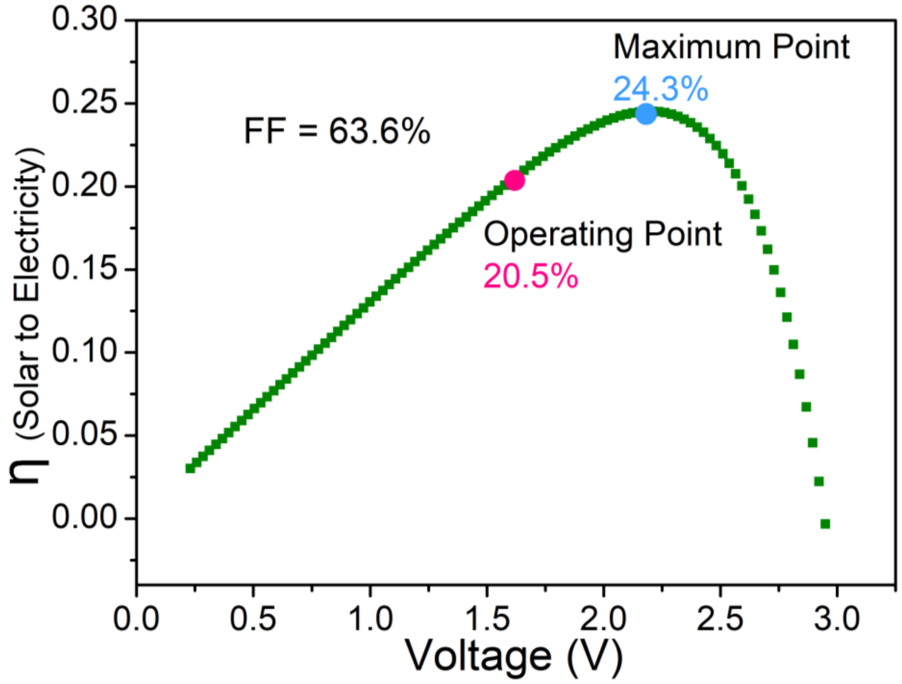

Figure S19. The solar to electricity conversion efficiency of the silicon solar cell under 0.87-fold AM 1.5 (87.13 mW cm^{-2}) illumination provided by solar simulator.

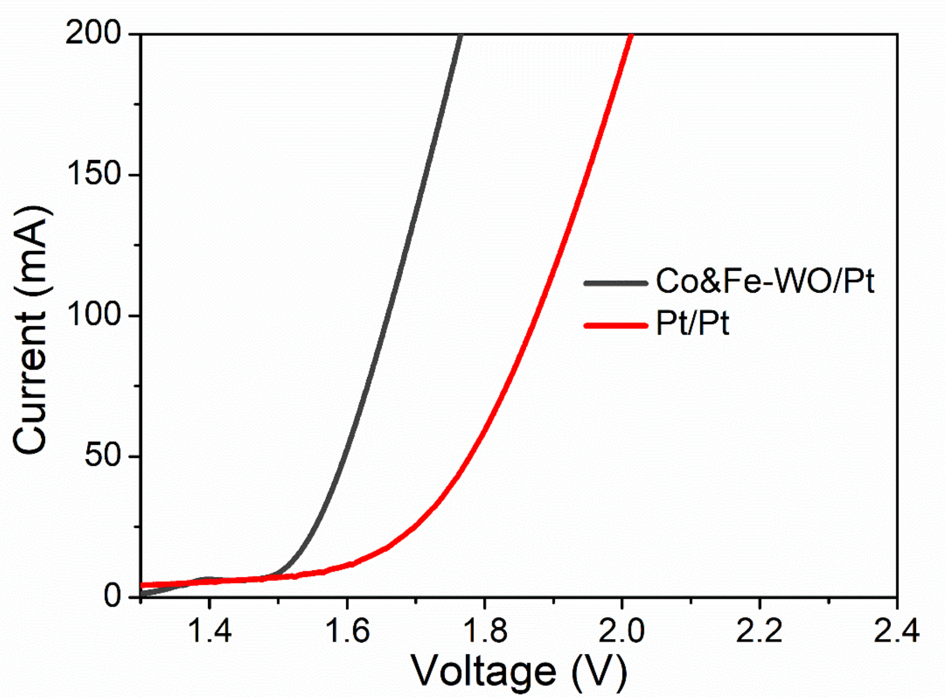

Figure S20. LSV curves for overall water-splitting of different electrode couples in a two-electrode configuration with the scan rate of 5 mV s^{-1}.

Table S1. ICP-MS results of the optimal Co&Fe-WO catalyst after desgassing.

Element	Composition (wt. %)
Co	1.53
Fe	3.17
W	68.12

Table S2. Recent W based and non-W based catalysts for oxygen evolution.

Category	Catalyst	Overpotential (mV) (J=10 mA cm^{-2})	Reference
W based	FeCoW oxyhydroxide/Au foam	191	*Science* **2016**, *352*, 333.
	$Mo_{(1-x)}W_xS_2$ Hollow Nanospheres	285	*ACS Appl. Mater. Interfaces* **2017**, *9*, 26066.
	WO_2 HN/Ni foam	300	*J. Mater. Chem. A* **2017**, *5*, 9655.
	N-WC	310 (0.5M H_2SO_4)	*Nat. Commun.* **2018**, *9*, 924.
	CoOx/WOx/GE	436	*Electrochim. Acta* **2017**, *224*, 551.
Non-W based	N-$(Ni,Fe)_3S_2$/NIF	167	*J. Mater. Chem. A* **2018**, *6*, 5592.
	core-shell NiFeCu	180	*Nat. Commun.* **2018**, *9*, 381.
	MoS_2/FNS/FeNi foam	204	*Adv. Mater.* **2018**, *30*, 1803151.
	Single-Atom Au/NiFe LDH	210	*J. Am. Chem. Soc.* **2018**, *140*, 3876.
	NiFe Prussian blue analogue (PBA)	258	*J. Am. Chem. Soc.* **2018**, *140*, 11286.
	FeCo Alloys and Co_4N Hybrid	280	*Adv. Mater.* **2017**, *29*, 1704091.
	core-shell ZIF-8@ZIF-67	310	*J. Am. Chem. Soc.* **2018**, *140*, 2610.
	G-Ni_4Fe/GF	310	*Adv. Energy Mater.* **2018**, *8*, 1800403.
	$NiSe_2$ nanosheets	330	*Adv. Mater.* **2017**, *29*, 1701687.
	NiO/Ni_3N	350	*Adv. Mater.* **2018**, *30*, 1803367.

Table S3. The proportion of different W species extracted from the W 4f XPS spectra.

Catalyst	W^{5+}	W^{6+}
WO	55.3%	44.7%
Co&Fe-WO	66.9%	33.1%

Table S4. The proportion of different O species extracted from the O 1s XPS spectra.

Catalyst	WO	Co-WO	Co&Fe-WO	Fe-WO
O3/(O1+O2)	0.2152	0.9021	1.2075	1.6580
